# Supplementary material for: FIKK1, a member of the FIKK kinase family, phosphorylates VAR2CSA and regulates adhesion of Plasmodium falciparum-infected erythrocytes to the placental receptor CSA
Source: PLoS Pathog. 2026 Jul 29;22(7):e1014108. doi: 10.1371/journal.ppat.1014108 (PMC13436824; doi:10.1371/journal.ppat.1014108)
Supplement: Supplementary file 1 [file ppat.1014108.s006.pdf]

S1B_raw_images

Fig 1B

B

Stained free gel before blotting

- WB Membrane cut in 2 parts after transfer
- Upper and bottom parts incubated with 2 different antibodies

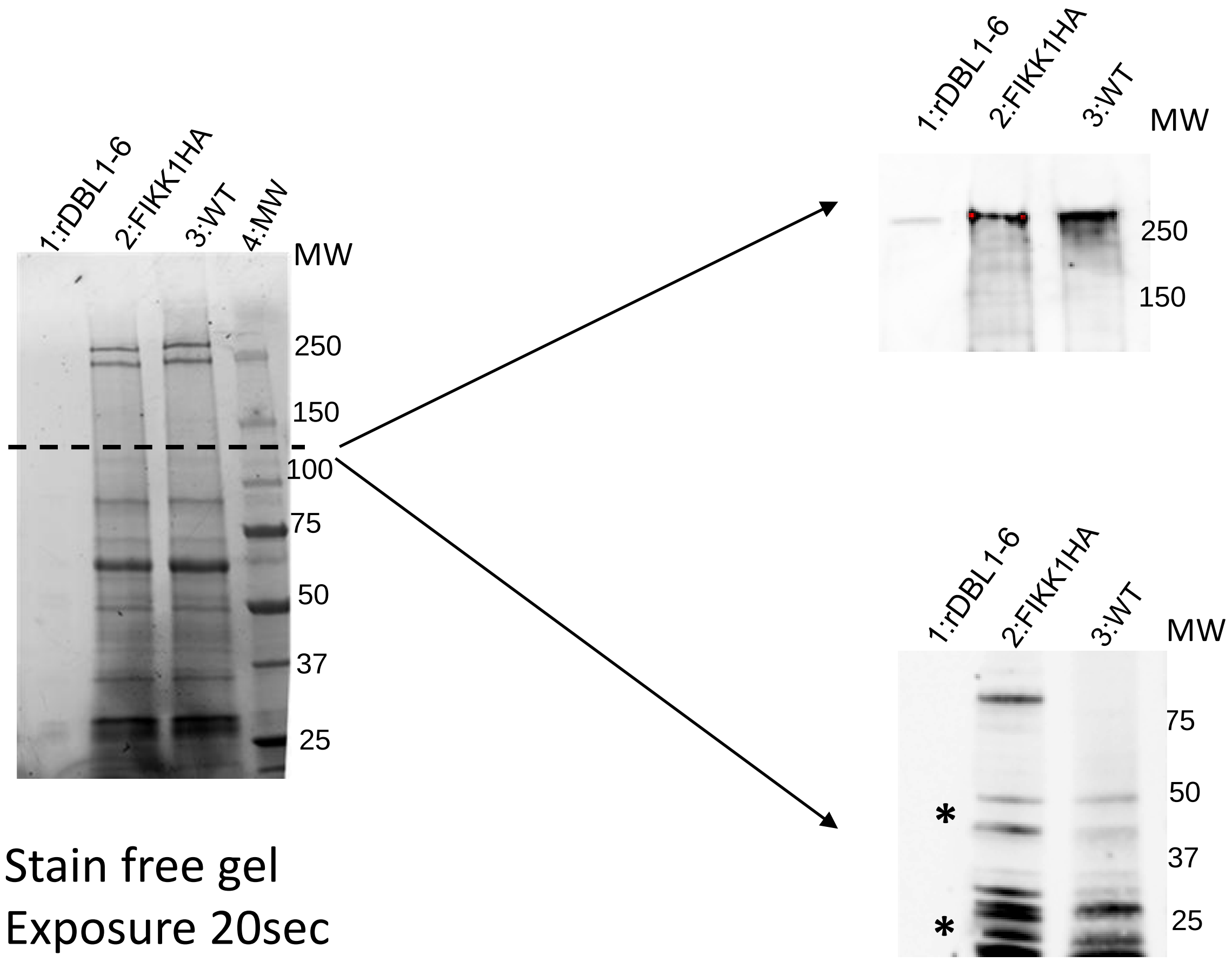

Stain free gel
Exposure 20sec

Western Blot

Western Blot

Aspecific bands * were obtained with
this anti HA antibody

Chemi Doc MP Imaging system

S2A_raw_images

Fig 2A

- Uncropped original WB **Membrane** was cut in 2 parts after transfer
- Upper and bottom parts incubated with 2 different antibodies

Stained free gel before blotting

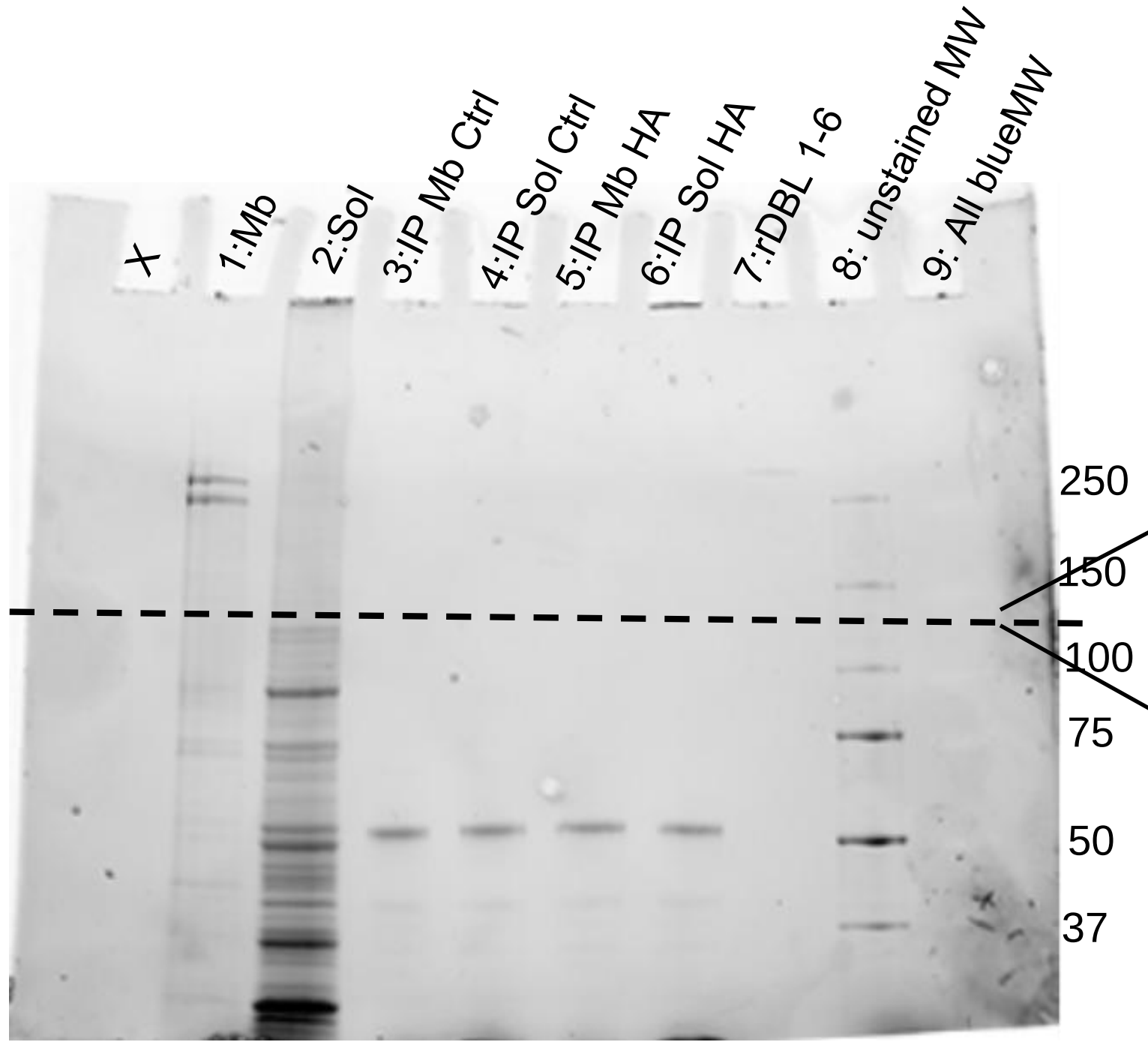

Stain free gel
Exposure 10sec

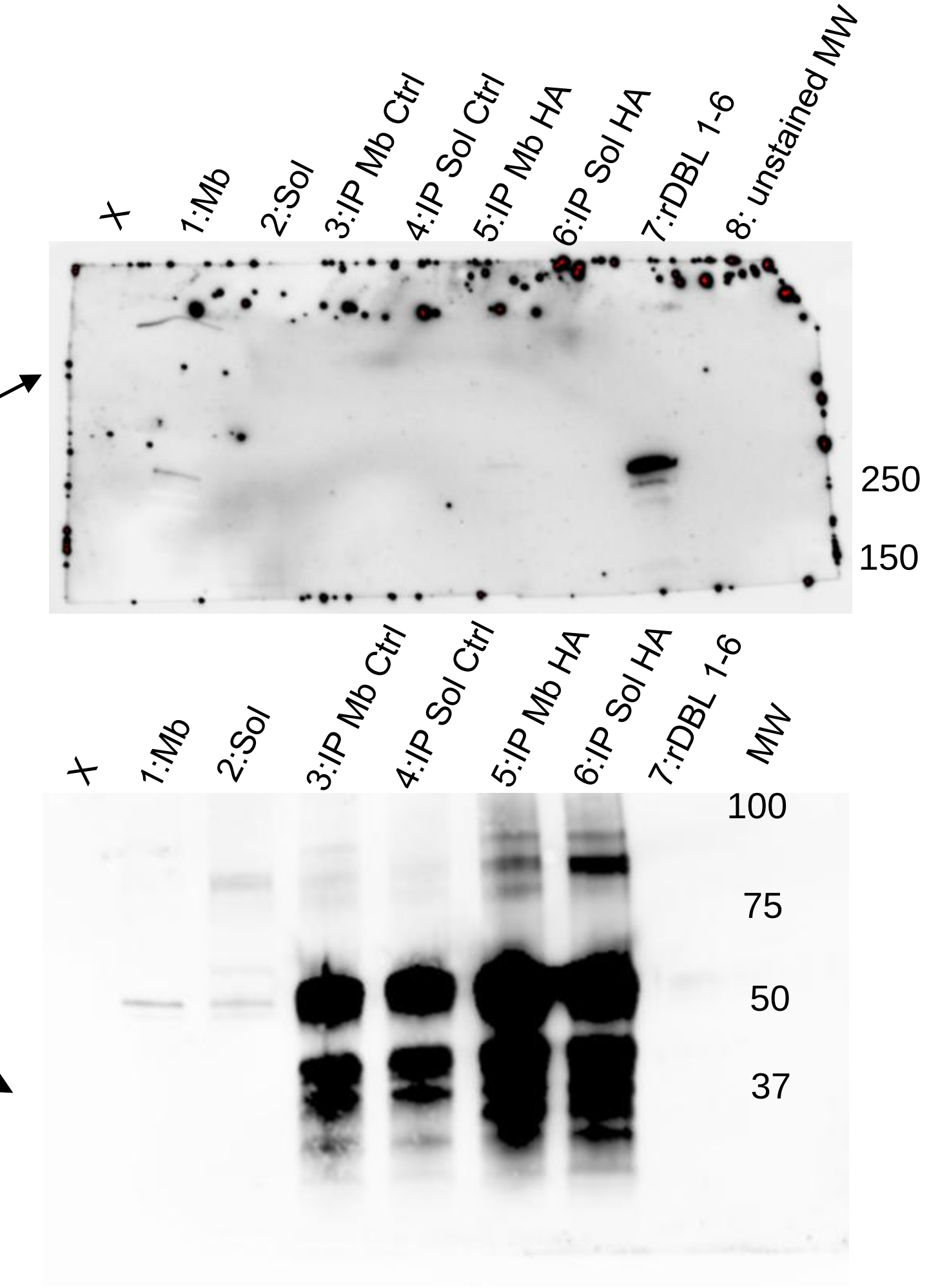

Chemi Doc MP Imaging system

S2B_raw_images

Fig 2B

Chemi Doc MP Imaging system

B

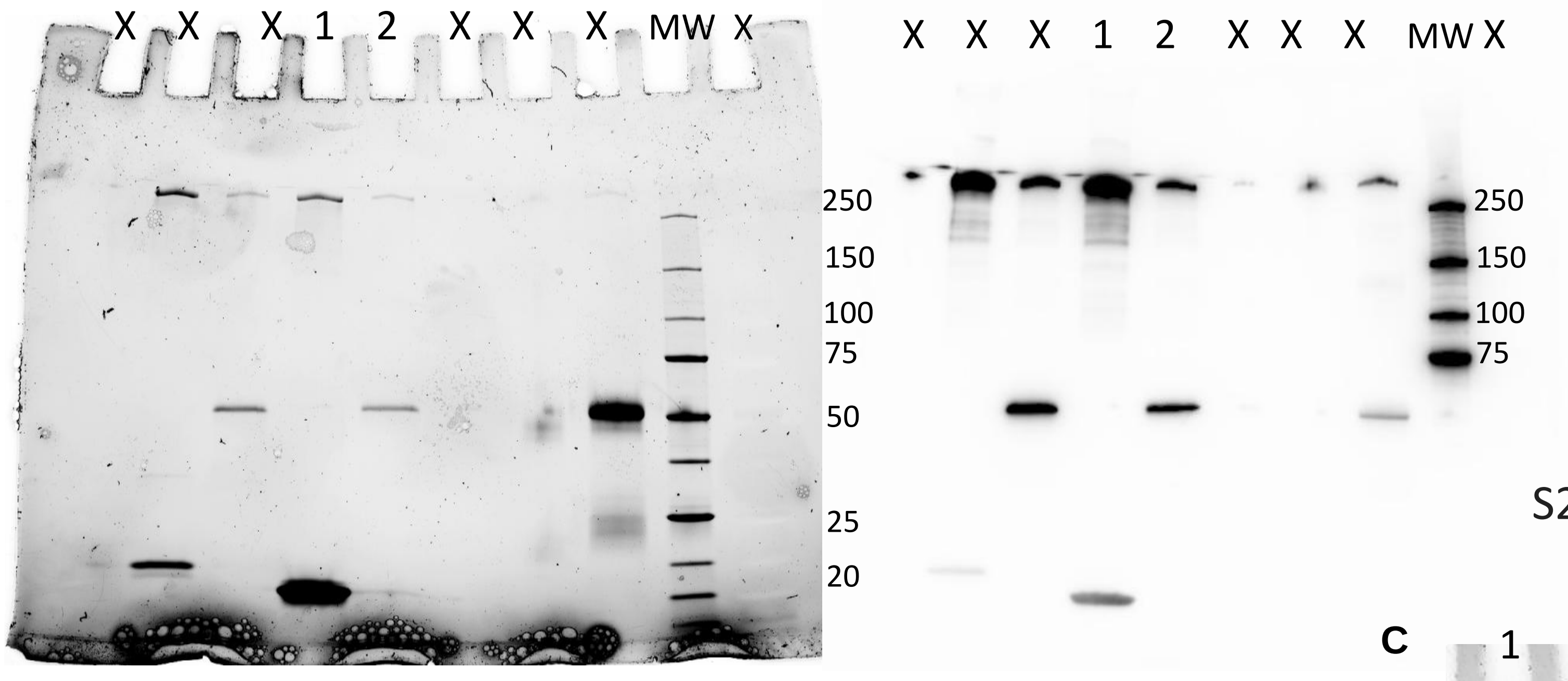

Stain free gel
Exposure 20sec

Western Blot

S2C_raw_images

Fig 2C

C

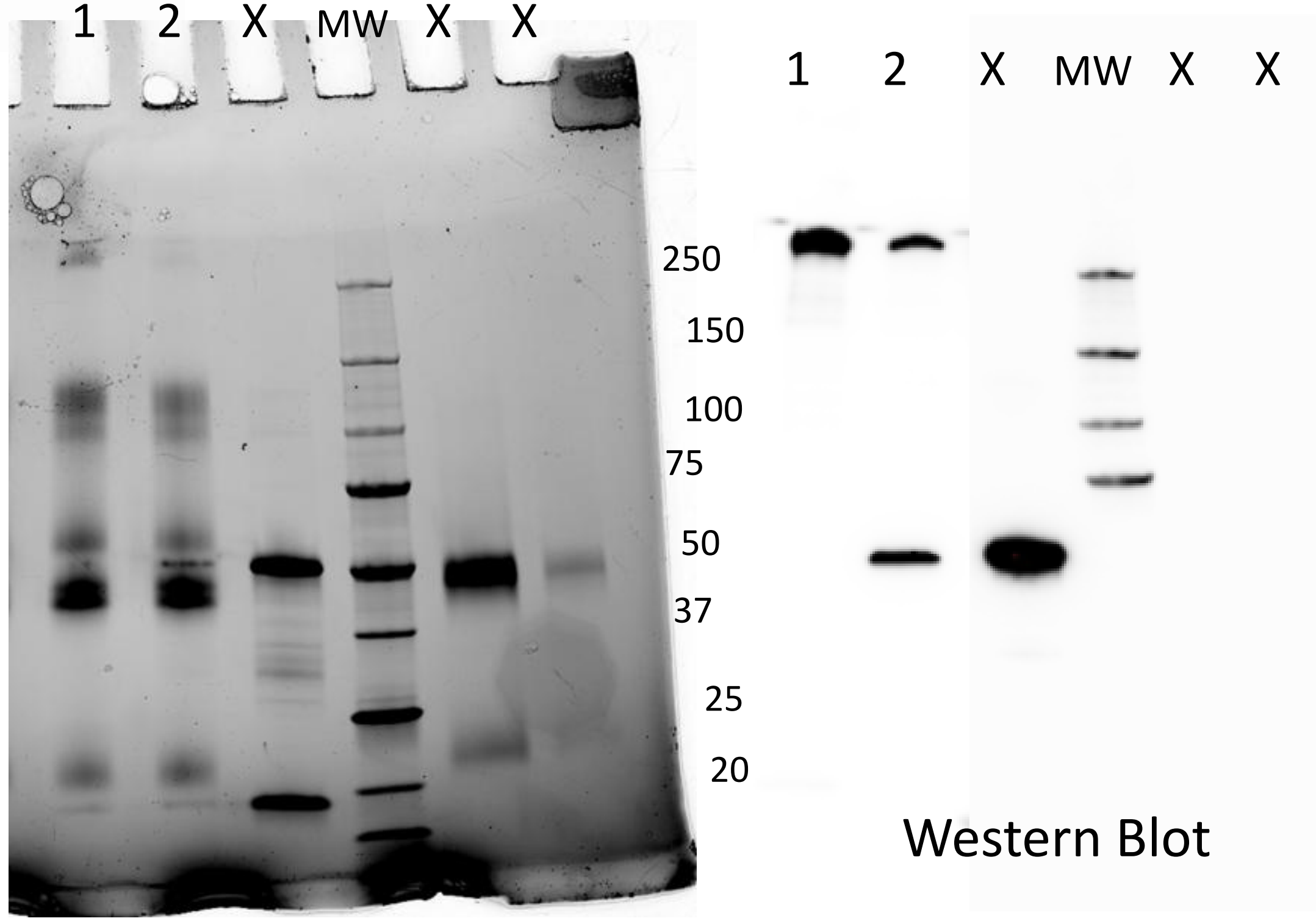

Stain free gel
Exposure 20sec

Western Blot

B

Stained free gel before blotting

- Uncropped original WB **Membrane** was cut in 2 parts after transfer
- Upper and bottom parts incubated with 2 different antibodies

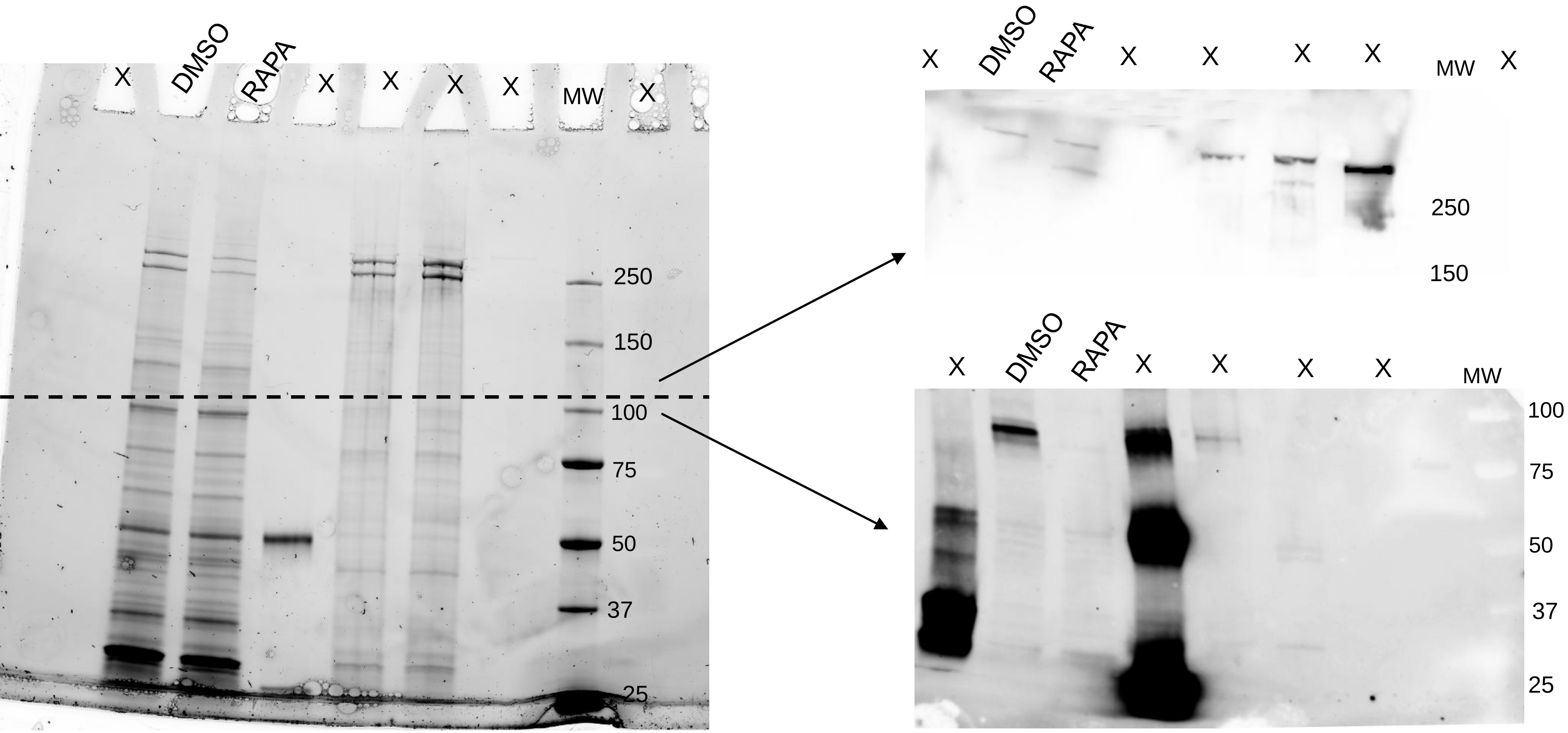

This WB is not shown in this manuscript

Western Blot

Stain free gel
Exposure 20sec

Chemi Doc MP Imaging system

S3D_raw_images Fig 3D

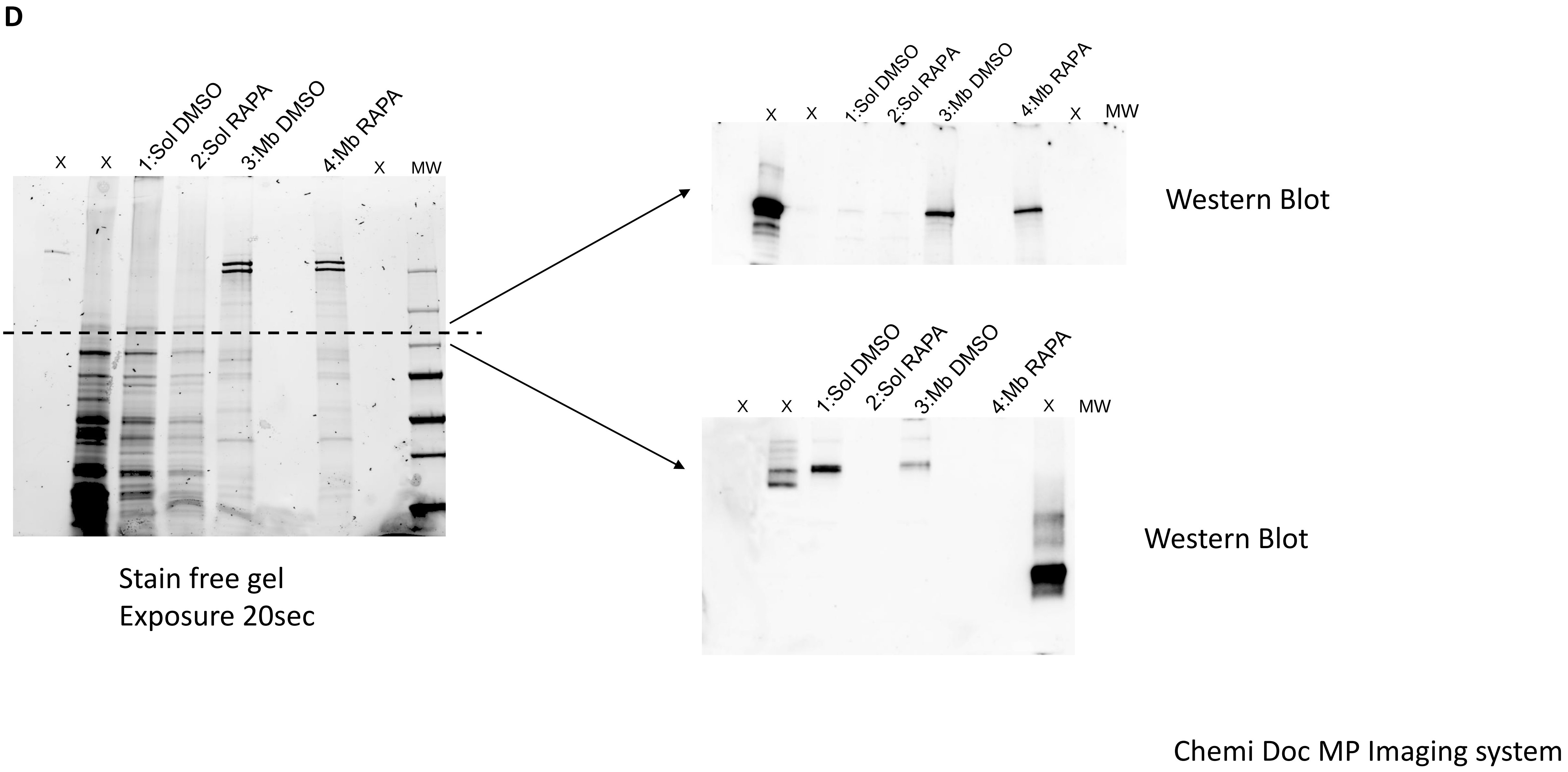

S5A_raw_images

Fig 5A and 5B

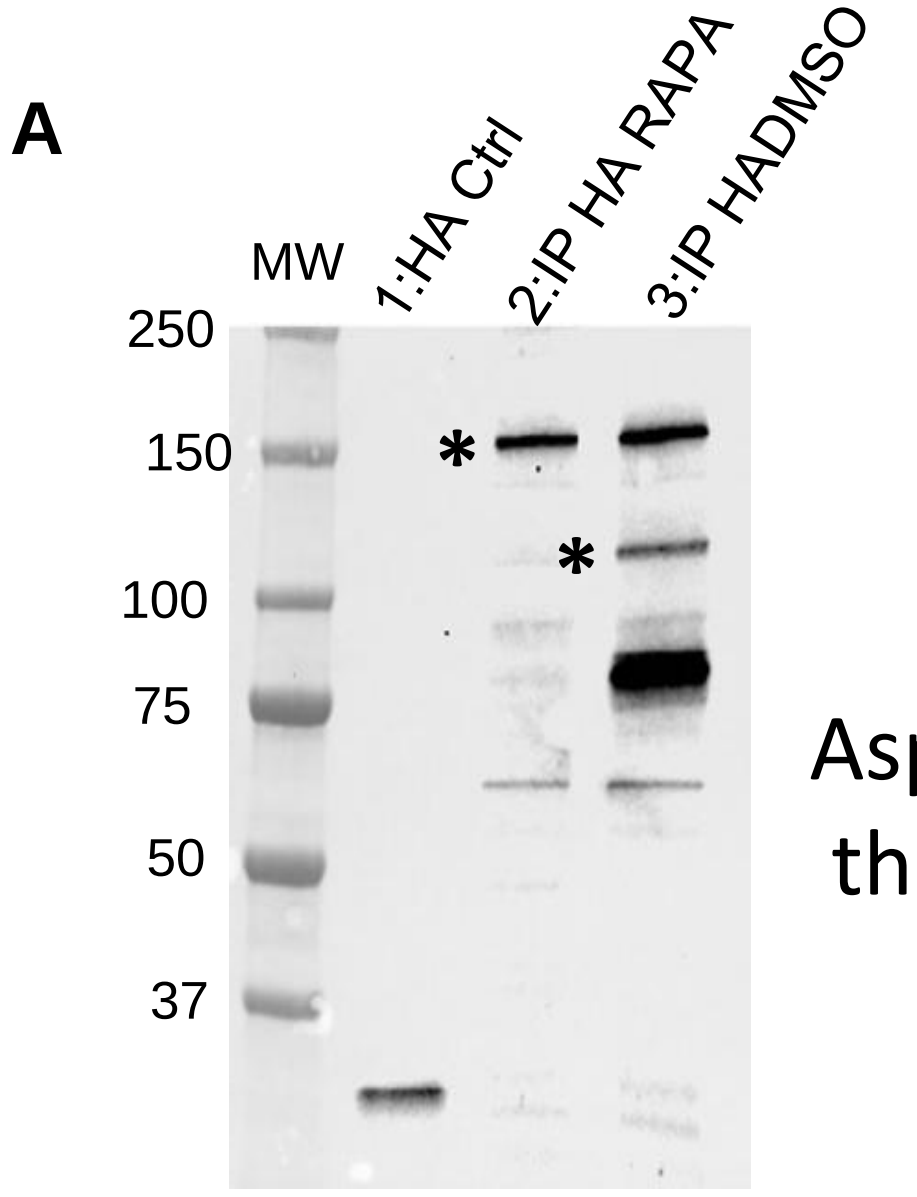

Aspecific bands * obtained with this anti HA antibody

Western Blot

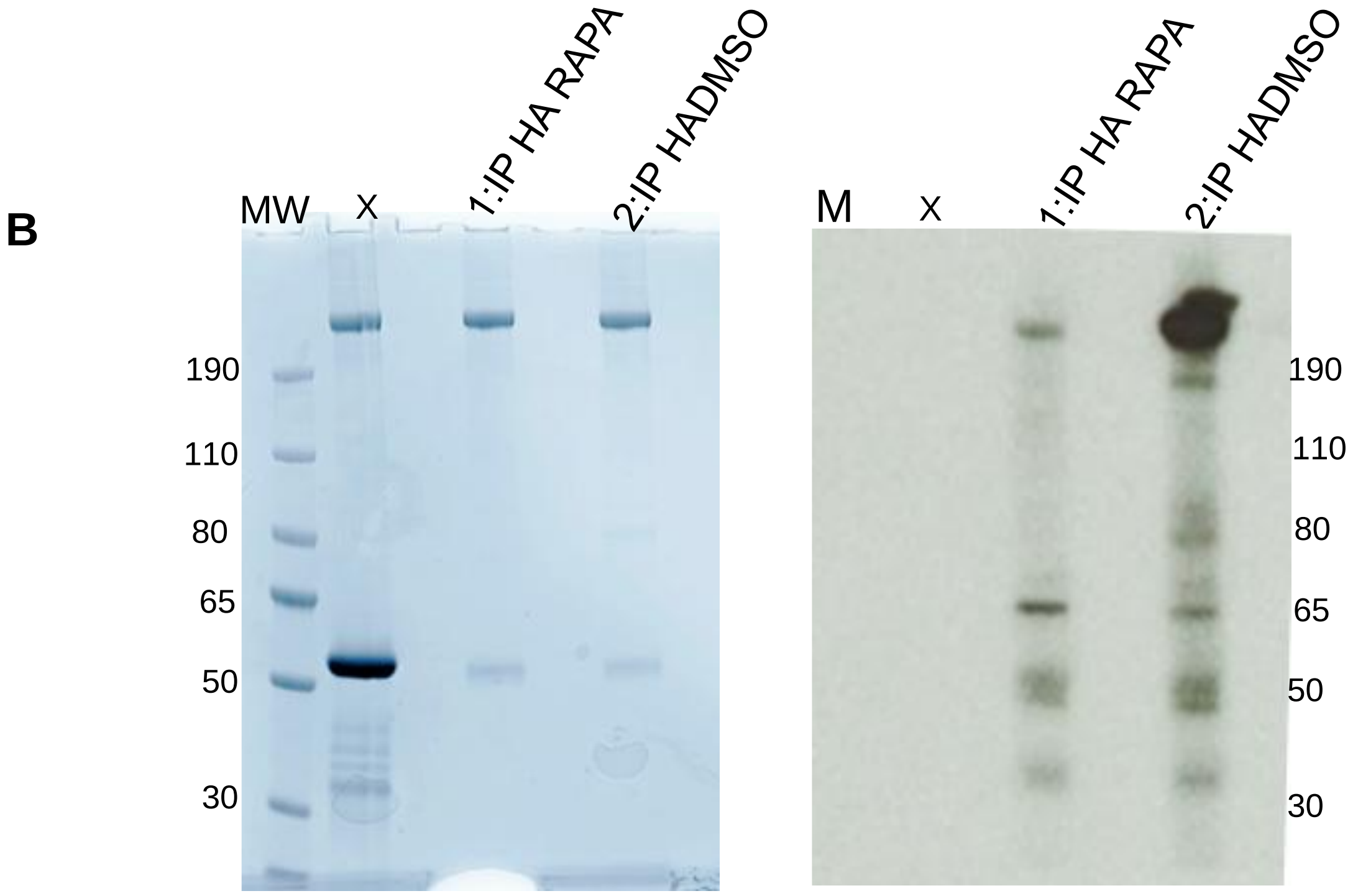

Coomassie gel followed by autoradiography
Exposure 16h

S5C_raw_images

Fig 5C

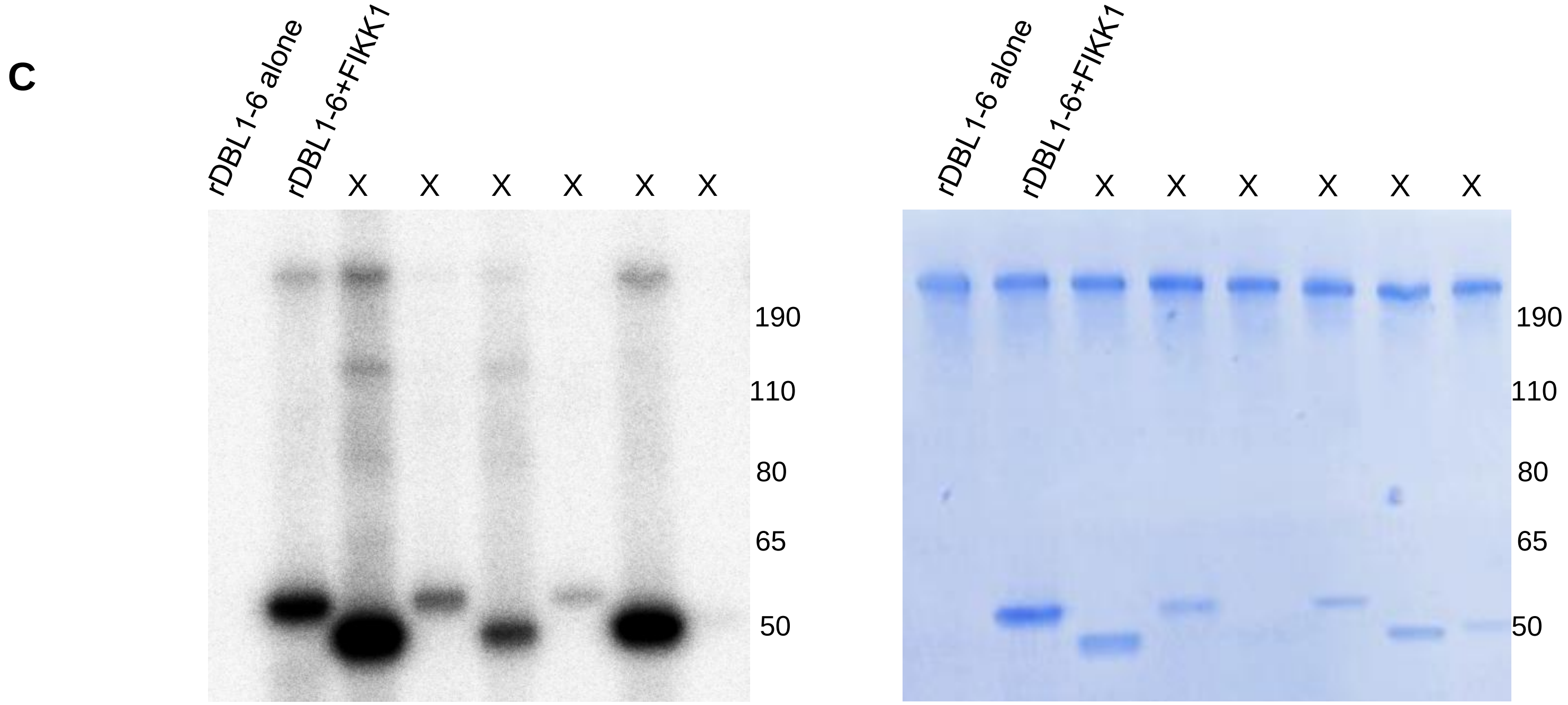

Coomassie gel followed by autoradiography
Exposure 16h

Figure 5 D

S5D_raw_images

D

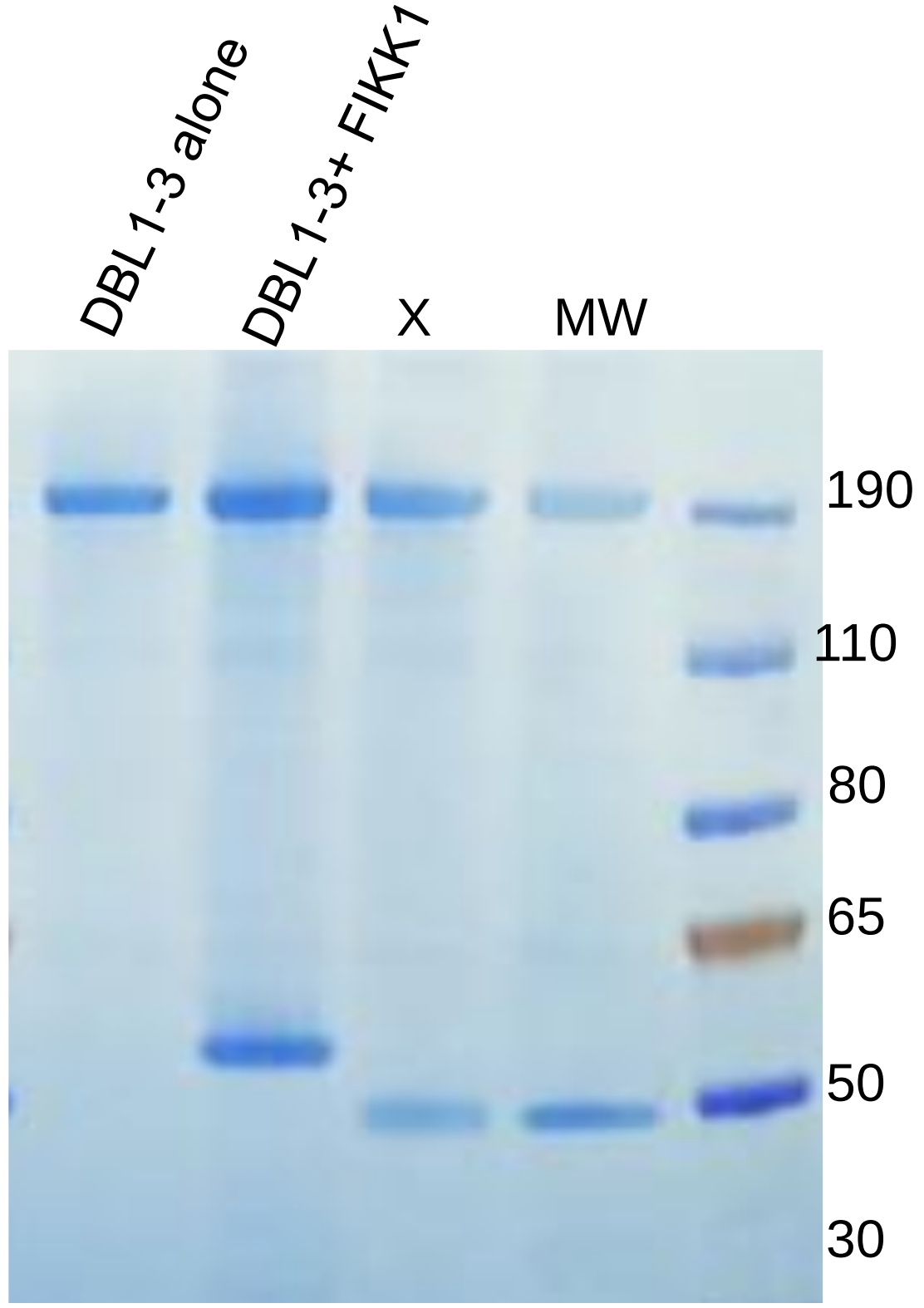

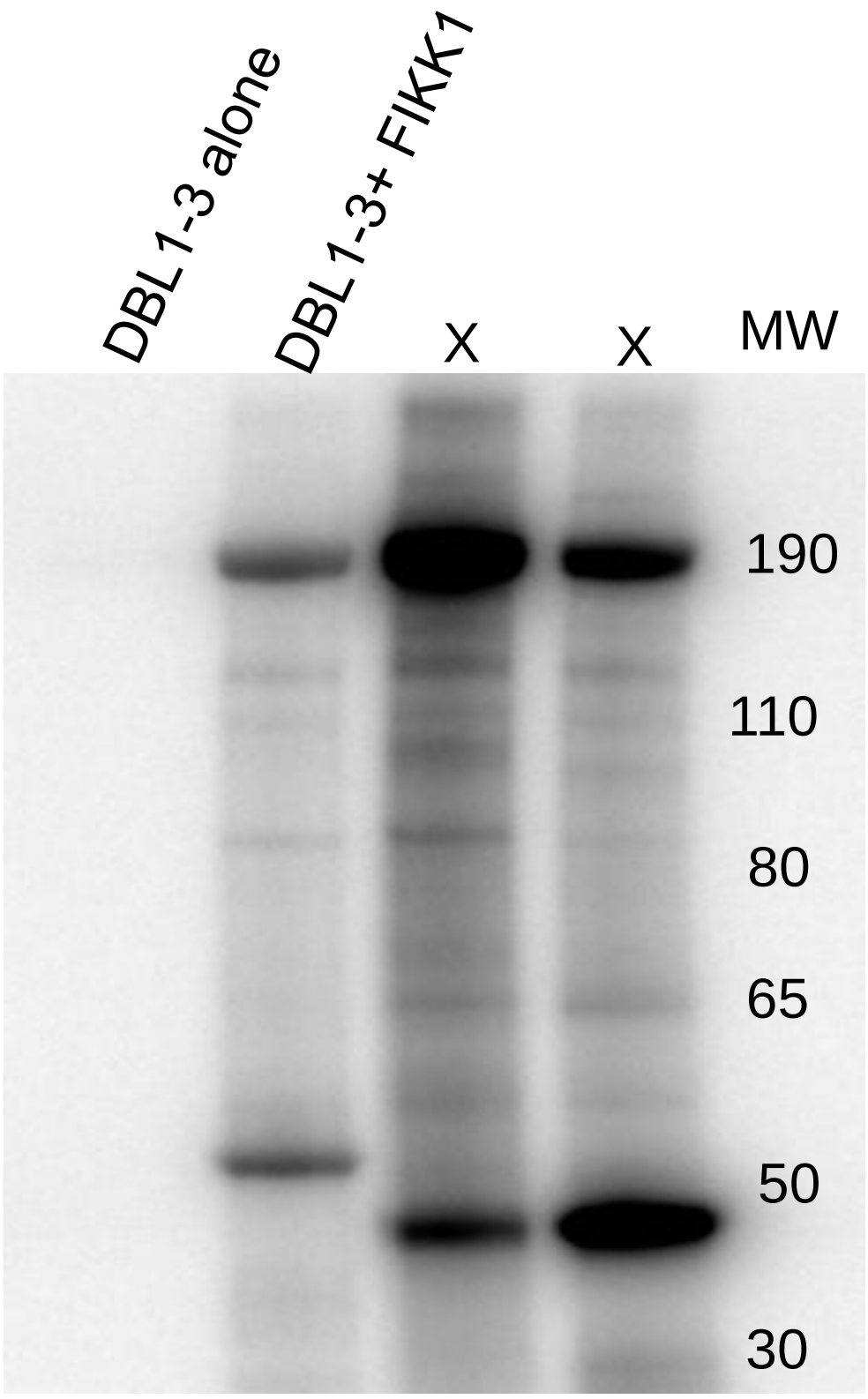

Coomassie gel followed by autoradiography
Film exposure 16h

S5E_ and S5F_ raw_images

Figure 5 E and 5F

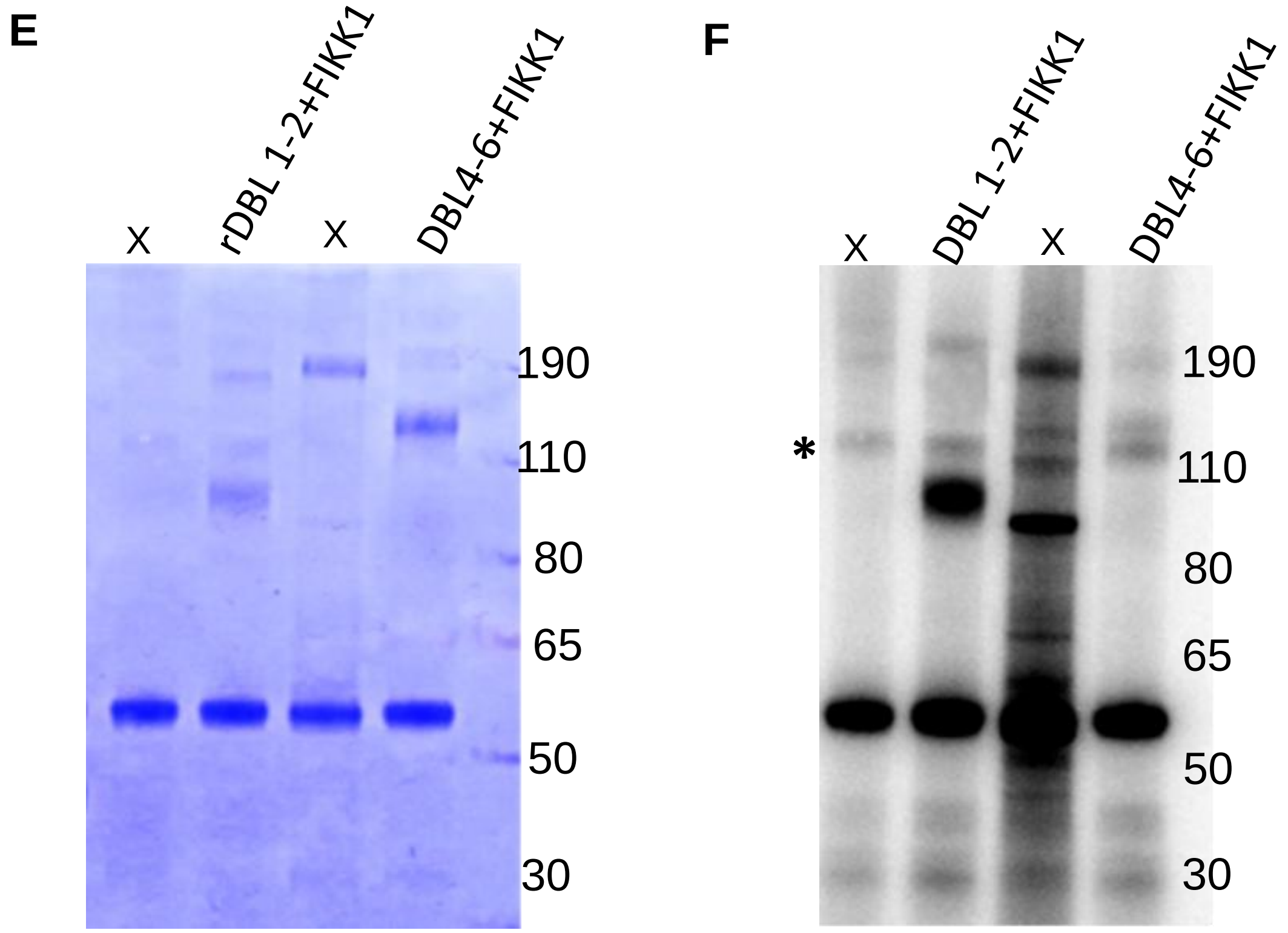

One aspecific band * is always present even in control (no substrate)

Coomassie gel followed by autoradiography
Film exposure 16h

S6B_ raw_images

Figure 6B

B

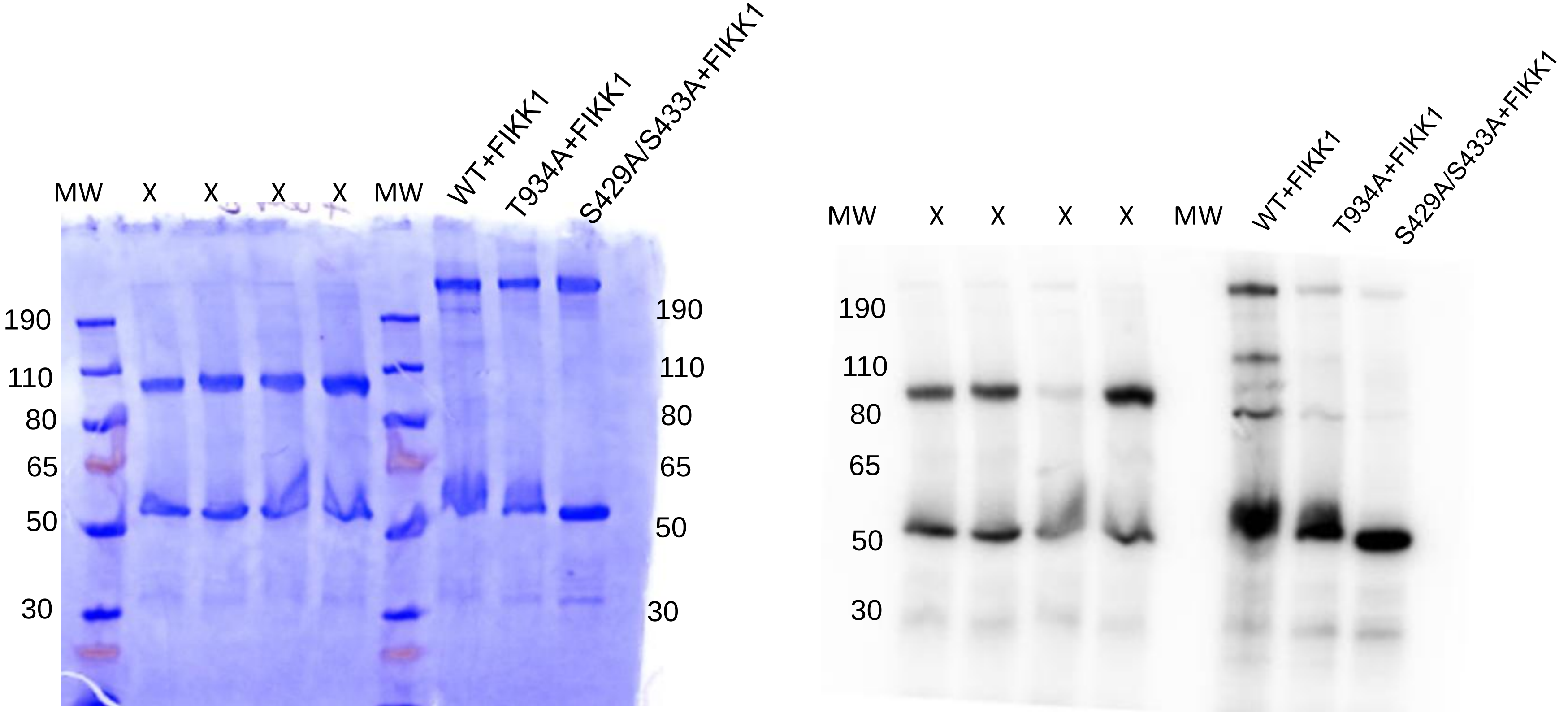

Coomassie gel followed by autoradiography
Exposure 16h
