# Supplementary material for: FIKK1, a member of the FIKK kinase family, phosphorylates VAR2CSA and regulates adhesion of Plasmodium falciparum-infected erythrocytes to the placental receptor CSA
Source: PLoS Pathog. 2026 Jul 29;22(7):e1014108. doi: 10.1371/journal.ppat.1014108 (PMC13436824; doi:10.1371/journal.ppat.1014108)
Supplement: Supplementary file 2 [file ppat.1014108.s009.docx]

Paris, 18/06/2026

**From:** Benoît Gamain and Dominique Dorin-Semblat

**To:** PLOS Pathology Editorial Board

Dear Editor,

You’ll find below our answers to the reviewers’ comments

With kind regards and many thanks for your consideration.

Best regards,

Benoît Gamain and Dominique Dorin-Semblat on behalf of all authors

<https://cimiparis.fr/>

**Centre d’Immunologie et des Maladies Infectieuses**
CIMI – (Sorbonne Université - Inserm U1135 - CNRS EMR8255)
Faculté de médecine Sorbonne Université

91 boulevard de l’hôpital | 75013 Paris
France

**Part I – Summary**

Please use this section to discuss strengths/weaknesses of study, novelty/significance, general execution and scholarship.

**Reviewer #1:** In this manuscript, Dorin Semblat and colleagues show that the well-known P. falciparum adhesin VAR2CSA can be phosphorylated by the plasmodial kinase FIKK1. Using a FIKK1-HA conditional knockout transgenic line, recombinant proteins and phosphorylation assays, they show that phosphorylation by FIKK1 increases the binding of VAR2CSA to the placental receptor CSA. The authors propose that FIKK1 contributes to placental malaria virulence through RBC adherence in the placenta mediated by VAR2CSA phosphorylation.
These findings are interesting and relevant for the malaria community. The experiments performed and reported were thorough and convincing. In most cases, data and methods were clearly explained. The data provide the evidence needed for the authors to make their conclusions. I only have some minor points or questions that should be addressed.

We thank Reviewer 1 for her/his positive review on the manuscript.

**Reviewer #2:** The manuscript by Dorin-Semblat et al provides *in vitro* evidence to delineate the function of FIKK1 in phosphorylating VAR2CSA which impacts cytoadhesion property to CSA. The work described here follows a similar line of work recently published by the authors on VAR2CSA phosphorylation by PfCK2. This is a well-planned study that supports the conclusions drawn. The authors are aware of the limitations of the conclusions drawn from in vitro studies and refrained from over-interpreting their results. I recommend publication of this work with some modifications.

**Specific comments:**

Conclusions of this study are derived from orthogonal experiments that include, co-localization, co-IP, in vitro binding experiments, and kinase assays. Importantly, the authors demonstrate that the knockout of FIKK1 causes reduction in CSA adhesion while VAR2CSA expression remains unchanged. Authors recognize the possibility of another protein kinase (PfCK2?) co-immunoprecipitating that may contribute to VAR2CSA phosphorylation. Mass spectrometric analysis of the IP could have ruled out that possibility. Additionally, phosphoproteomic analysis of VAR2CSA in wild type vs FIKK1 cKO would have strengthened the conclusion.

We thank reviewer # 2 for these constructive and specific comments. Regarding the possibility that another kinase like PfCK2 could be coimmunoprecipitated with VAR2CSA and contribute to VAR2CSA phosphorylation: although we showed in our previous manuscript (Dorin Semblat, Plos pathogens 2025) that PfCK2 or HuCK2a bind **in *vitro*** to VAR2CSA, we did not detect PfCK2 in the Immunoprecipitates obtained with anti VAR2CSA antibodies from membrane fractions of infected RBCs.

We agree with reviewer #2 that phosphoproteomic analysis of VAR2CSA in wild type vs FIKK1 cKO would have strengthened the conclusion. We indeed tried to perform this experiment but failed due to the lack of material and/or due to the lability of the phosphosites. Although we were able to recover peptides from most of the VAR2CSA protein sequence, the number of phosphopeptides, even in the wild-type extract, was too low (or absent to enable us to draw any conclusions.

We also tried to develop a phosphor-specific antibody against pT934, but didn’t succeed in obtaining a specific antibody.

It is not clear if there are any phenotypic change during the induction by rapamycin.

In all 3 independent FIKK1 KO induction (Rapamycin-mediated) experiments performed, we did not observe any change in the growth or in the morphology of the parasite **after 72 hours of induction**. Our observations confirm the previously published data from the Treeck lab who compared the growth rates of DMSO- and RAP-treated FIKK1 cKO line along with the NF54::DiCre parental line by flow cytometry over 5 days. No significant difference was observed for FIKK1 over this time period (ref 24 in the manuscript). The only observed phenotypic change is a reduction in adhesion to CSA.

In Fig 6B, there are residual phosphorylation in DBL1-6 mutants, suggesting additional phosphosites may be involved.

We agree with this suggestion. Indeed, as shown in Table 1, residues other than T934 and S429 are phosphorylated by FIKK1. However, based on Fig6B, the strong decrease of phosphorylation signal suggests that these 2 residues are predominantly phosphorylated by FIKK1.

This is reflected in the following sentence page 12 lines 300-303 : “Interestingly, we observed a radiolabeling decrease of the VAR2CSA DBL1-6 mutant proteins compared to the DBL1-6 wild-type substrate **(Fig 6B left panel)** suggesting that T934 and S429 could be predominantly phosphorylated residues by the FIKK1.”

There is a possibility that FIKK1 can mediate changes in adhesion through its functions at the erythrocyte membranes rather than exclusively through VAR2CSA phosphorylation. Although the data presented in the manuscript supports the conclusions drawn, a robust discussion on other possibilities, such as changes erythrocyte cytoskeleton causing remodeling RBC membrane architecture, would improve the manuscript.

We agree with the reviewer and added the following paragraph at the end of the discussion : “While the data presented in our manuscript support the role of FIKK1 in regulating IEs adhesion to CSA through VAR2CSA phosphorylation, we cannot exclude that other mechanisms such as changes in erythrocyte cytoskeleton protein phosphorylation affecting IEs rigidity or knob structures could also contribute to the reduction in adhesion. Indeed, it was previously shown that FIKK1 is implicated in the phosphorylation of red blood cell adducin at position S726 [24]. In line with this hypothesis, a targeted disruption of the gene encoding FIKK4.2 (FIKK4.2) dramatically alters the parasite’s ability to remodel the red blood cells surface making the KO cell line less rigid and less adhesive, despite an unchanged level of antigenic variant PfEMP1 [23]. Membrane fluctuation analysis by flicker spectroscopy or in vitro microsphiltration assays [24] would allow to assess the potential role of FIKK1 in rigidity of infected erythrocytes”. This is ongoing work in the Treeck lab but goes beyond the scope of this manuscript.

Reviewer #3: The current manuscript by Dorin-Semblat et al shows that the P. falciparum kinase phosphorylates the parasite ligand var2csa increasing the parasite binding affinity to csa. The study is elegantly done using a combination of molecular tools, including knockout parasites, pull down and phosphorylation assays on parasite lysates and recombinant proteins, proteomics approaches and parasite binding studies. The authors show that the phosphorylation residues on the N-terminal multidomain region of the var2csa, and that preventing phosphorylation significantly reduces the parasite binding to csa. The study is sound, logical and experiments were well controlled. Although I consider that no additional experiments are needed for publication, there were some points that would strengthen the manuscript. Although they could be considered minor, these suggestions would significantly improve the clarity of the manuscript.

We thank reviewer # 3 for her very positive review

**Part II – Major Issues: Key Experiments Required for Acceptance**
Please use this section to detail the key new experiments or modifications of existing experiments that should be absolutely required to validate study conclusions.

Generally, there should be no more than 3 such required experiments or major modifications for a "Major Revision" recommendation. If more than 3 experiments are necessary to validate the study conclusions, then you are encouraged to recommend "Reject".

Reviewer #1: NA

Reviewer #2: None in particular.

Reviewer #3: (No Response)

**Part III – Minor Issues: Editorial and Data Presentation Modifications**
Please use this section for editorial suggestions as well as relatively minor modifications of existing data that would enhance clarity.

**Reviewer #1:** My main comment is that the authors identified several phosphosites (such as S429, S433) as phosphorylated by FIKK1, however they were also shown to be phosphorylated by PfCK2 in their previous paper (Dorin Semblat, Plos pathogens 2025). How do the authors explain these results? Do they think that these residues are phosphorylated by both kinases? They should discuss this point.

This is a good point that other kinases could phosphorylate the same residues. Although the reviewer is right that S433 has been shown to be phosphorylated by PfCK2a, S429 is not phosphorylated *in vitro* by PfCK2a. Also, S433 is the only phosphoresidue detected in MS analysis of rVAR2CSA DBL1-3 prior FIKK1 addition (Table1). It is therefore difficult to ascertain whether or not there is an additional phosphorylation on S433 by FIKK1 according to the MS data.

The selection for a clone positive for VAR2CSA expression at the IE surface is not clearly explained. It would help the reader to better explain their protocol.

The objective was not to select parasites expressing VAR2CSA on their surface; rather we wanted to ascertain that the parasites provided by the Treeck lab already expressing VAR2CSA at high levels was a homogenous population, which was necessary for our functional studies. The transgenic population was then recloned by limiting dilution and genotyping was performed on several clones. Clear absence of WT signal and correct integration in the tested clones shown by PCR (cf supplementary figures) as well as VAR2CSA expression were the critical requirement for selecting the clones (clone F5 in this study) to pursue all the experiments.

In the text, page 7 (lines 135 – 137) we indicated: To ascertain homogeneity of the parasites, the fikk1 cKO transgenic cell line was recloned by limiting dilution and the F5 clone was characterized by flow cytometry for VAR2CSA expression.

Interestingly, Figure 5B suggests that FIKK1 phosphorylates the endogenous VAR2CSA (band indicated with a *). However, it would be more convincing to load immuno-conjugated material (DMSO and RAPA-treated) incubated with ATP32 alone to confirm this observation.

We agree with the reviewer’s remark that an additional control experiment could be done to confirm that the radiolabeled band corresponds to the endogenous VAR2CSA. We have therefore softened the statement as follows:

“Interestingly, we observed a radiolabeled band with a high molecular weight (>300kDa) (indicated with a *) suggesting that endogenous VAR2CSA could be co-immunoprecipitated and phosphorylated by FIKK1HA”

Instead of:

“Interestingly, we observed a radiolabeled band with a high molecular weight (>300kDa) corresponding to the size of full-length VAR2CSA (indicated with a *) just above the labelled recombinant VAR2CSA extracellular region”

We also modified the following sentence in the discussion:

“Additionally, we observed a high molecular weight radiolabeled band migrating just above the radiolabeled ectodomain DBL1-6 that could correspond to the endogenous full length VAR2CSA co-immunoprecipitated with FIKK1-HA” with :

“Additionally, we observed a high molecular weight radiolabeled band migrating just above the radiolabeled ectodomain DBL1-6 that could correspond to the endogenous full length VAR2CSA or any other cytoskeleton or membrane bound protein with a high MW coimmunoprecipitating and phosphorylated by FIKK1-HA”.

There is a typo line 242:

The typo has been removed

Reviewer #2: It is suggested to include alternative possibilities of FIKK1-mediated changes in adhesion.

We agree with the reviewer and added the following paragraph at the end of the discussion :

“While the data presented in our manuscript support the role of FIKK1 in regulating IEs adhesion to CSA through VAR2CSA phosphorylation, we cannot exclude that other mechanisms such as changes in erythrocyte cytoskeleton protein phosphorylation affecting IEs rigidity or knob structures could also contribute to the reduction in adhesion. Indeed, it was previously shown that FIKK1 is implicated in the phosphorylation of red blood cell adducin at position S726 [24]. In line with this hypothesis, a targeted disruption of the gene encoding FIKK4.2 (FIKK4.2) dramatically alters the parasite’s ability to remodel the red blood cells surface making the KO cell line less rigid and less adhesive, despite an unchanged level of antigenic variant PfEMP1 [23]. Membrane fluctuation analysis by flicker spectroscopy or in vitro microsphiltration assays [24] would allow to assess the potential role of FIKK1 in rigidity of infected erythrocytes”.

Reviewer #3: - Quantification of immunofluorescences: I would suggest for good scientific practice and for better representation of experimental variability that immunofluorescences in the manuscript are quantified. This is especially important for the images shown in Figure 1C in which co-localization between var2csa and SBP1 seem to be higher than with FIKK1HA. How many iRBC were quantified in this experiment? Similarly, quantification of Figure 3A is missing, and in Figure 3E I suggest to move the FACS quantification to the main figure, as this is the most representative experiment of the phenotype. Likewise, I would clearly show that Figure 4A and B correspond to the same experiment.

We thank the reviewer for her suggestion. The immunofluorescence assays of infected erythrocytes images are representative from at least 3 independent experiments with similar results. Although it would provide an extra layer of information, quantifying the immunofluorescence would take quite some time since it would require us to undertake a *de novo* IFA campaign in order to acquire large sets of images. Therefore, we prefer not to quantify the immunofluorescence. On the other hand, we fully agree with the reviewer to move the FACS quantification to the main figure (Fig 3E) since this is the most representative experiment of the phenotype.

- I would suggest to indicate the wall shear stress instead of flow rate, as the later is dependent on the dimensions of the channel/flow chamber. If the authors use an IBIDI set up, a conversion should be available in the vendors website.

We have now indicated the wall shear stress instead of flow rate throughout the manuscript.

- Clarity of figures: The figures in the manuscript are not self-explanatory and this impacts the reading of the manuscript. Simple experimental schematics or labeling the protein gels and western blot lines, instead of just numbering them will be helpful for the reader.

We tried to simplify the labeling of the gels and western blot lines instead of numbering them.

- PfEMP1 terminology. The var2csa structure is not shown, which might make difficult the understanding certain sections of the manuscript for non var/PfEMP1 aficionados. This is especially important for Figures 5 and 6. Also, the authors use interchangeably the term extracellular domain of VAR2CSA and rDBL1.6, this could be confusing when different terms are used in the result section and in the figure.

We have checked carefully the manuscript and have now used rDBL1-6 throughout the manuscript. We also included a supplementary figure representing the different recombinant VAR2CSA proteins.

- Implications for future development of drugs. The unphosphorylated form of var2csa still presents cytoadhesion ?. Although the authors do not claim that a pan-FIKK inhibitor would prevent cytoadhesion, the reference in the text (line 407-409) appears a bit out of the blue. It could be good to add a couple of sentences to contextualize it with the results of the manuscript.

We added the following sentence to clarify this point: “Although in the case of KO FIKK1 IEs expressing VAR2CSA we observed *in vitro* a decrease of adhesion above 50%, we cannot extrapolate that a pan-FIKK inhibitor would completely block *in vivo* IEs adhesion and hence sequestration, unless other important VAR2CSA residues are targeted by members of the FIKK kinase family. A pan-FIKK inhibitor could also affect the rigidity, the knob-structures or the translocation of VAR2CSA on the IEs surface.
