# Supplementary material for: FIKK1, a member of the FIKK kinase family, phosphorylates VAR2CSA and regulates adhesion of Plasmodium falciparum-infected erythrocytes to the placental receptor CSA
Source: PLoS Pathog. 2026 Jul 29;22(7):e1014108. doi: 10.1371/journal.ppat.1014108 (PMC13436824; doi:10.1371/journal.ppat.1014108)
Supplement: Peer review history

# Peer review: Decision Letter 0

**DOI:** 10.1371/journal.ppat.1014108.r001

**Authors:** Tracey Lamb, Leann Tilley

21 May 2026

PPATHOGENS-D-26-00737

FIKK1, a member of the FIKK kinase family, phosphorylates VAR2CSA and regulates adhesion of Plasmodium falciparum-infected erythrocytes to the placental receptor CSA

PLOS Pathogens

Dear Dr. Gamain,

Thank you for submitting your manuscript to PLOS Pathogens. After careful consideration, we feel that it has merit but does not fully meet PLOS Pathogens's publication criteria as it currently stands. Therefore, we invite you to submit a revised version of the manuscript that addresses the points raised during the review process.

Please submit your revised manuscript by Jul 20 2026 11:59PM. If you will need more time than this to complete your revisions, please reply to this message or contact the journal office at plospathogens@plos.org. When you're ready to submit your revision, log on to https://www.editorialmanager.com/ppathogens/ and select the 'Submissions Needing Revision' folder to locate your manuscript file.

Please include the following items when submitting your revised manuscript:

* A letter that responds to each point raised by the editor and reviewer(s). You should upload this letter as a separate file labeled 'Response to Reviewers'. This file does not need to include responses to any formatting updates and technical items listed in the 'Journal Requirements' section below.

* A marked-up copy of your manuscript that highlights changes made to the original version. You should upload this as a separate file labeled 'Revised Manuscript with Track Changes'.

* An unmarked version of your revised paper without tracked changes. You should upload this as a separate file labeled 'Manuscript'.

If you would like to make changes to your financial disclosure, competing interests statement, or data availability statement, please make these updates within the submission form at the time of resubmission. Guidelines for resubmitting your figure files are available below the reviewer comments at the end of this letter.

As the corresponding author, your ORCID iD is verified in the submission system and will appear in the published article. PLOS supports the use of ORCID, and we encourage all coauthors to register for an ORCID iD and use it as well. Please encourage your coauthors to verify their ORCID iD within the submission system before final acceptance, as unverified ORCID iDs will not appear in the published article. Only the individual author can complete the verification step; PLOS staff cannot verify ORCID iDs on behalf of authors.

We look forward to receiving your revised manuscript.

Kind regards,

Leann Tilley

Academic Editor

PLOS Pathogens

Tracey Lamb

Section Editor

PLOS Pathogens

Sumita Bhaduri-McIntosh

Editor-in-Chief

PLOS Pathogens

orcid.org/0000-0003-2946-9497

Michael Malim

Editor-in-Chief

PLOS Pathogens

orcid.org/0000-0002-7699-2064

**Additional Editor Comments (if provided):**

The authors should pay particular attention to the Reviewer's suggestions for additional controls.

**Journal Requirements:**

If the reviewer comments include a recommendation to cite specific previously published works, please review and evaluate these publications to determine whether they are relevant and should be cited. There is no requirement to cite these works unless the editor has indicated otherwise.

1) Please ensure that the CRediT author contributions listed for every co-author are completed accurately and in full.

At this stage, the following Authors/Authors require contributions: Dominique Dorin-Semblat, Jean-Philippe Semblat, Heledd Eavis, Hugo Belda, Romain Hamelin, Clara-Eva Paquereau, Christian Doerig, Moritz Treeck, and Benoît Gamain. Please ensure that the full contributions of each author are acknowledged in the "Add/Edit/Remove Authors" section of our submission form.

The list of CRediT author contributions may be found here: https://journals.plos.org/plospathogens/s/authorship#loc-author-contributions

2) We do not publish any copyright or trademark symbols that usually accompany proprietary names, eg ©,  ®, or TM  (e.g. next to drug or reagent names). Therefore please remove all instances of trademark/copyright symbols throughout the text, including:

- ® on page: 27

- TM on page: 27.

3) Please upload all main figures as separate Figure files in .tif or .eps format. For more information about how to convert and format your figure files please see our guidelines:

https://journals.plos.org/plospathogens/s/figures

4) PLOS now requires that authors provide the original uncropped and unadjusted images underlying all blot or gel results reported in a submission’s figures or Supporting Information files. This policy and the journal’s other requirements for blot/gel reporting and figure preparation are described in detail at https://journals.plos.org/plosone/s/figures#loc-blot-and-gel-reporting-requirements and https://journals.plos.org/plosone/s/figures#loc-preparing-figures-from-image-files.

When you submit your revised manuscript, please ensure that your figures adhere fully to these guidelines and provide the original underlying images for all blot or gel data reported in your submission. See the following link for instructions on providing the original image data: https://journals.plos.org/plosone/s/figures#loc-original-images-for-blots-and-gels.

In your cover letter, please note whether your blot/gel image data are in Supporting Information or posted at a public data repository, provide the repository URL if relevant, and provide specific details as to which raw blot/gel images, if any, are not available. Email us at customercare@plos.org if you have any questions.

**Reviewers' Comments:**

Reviewer's Responses to Questions

**Part I - Summary**

Please use this section to discuss strengths/weaknesses of study, novelty/significance, general execution and scholarship.

Reviewer #1: In this manuscript, Dorin Semblat and colleagues show that the well-known P. falciparum adhesin VAR2CSA can be phosphorylated by the plasmodial kinase FIKK1. Using a FIKK1-HA conditional knockout transgenic line, recombinant proteins and phosphorylation assays, they show that phosphorylation by FIKK1 increases the binding of VAR2CSA to the placental receptor CSA. The authors propose that FIKK1 contributes to placental malaria virulence through RBC adherence in the placenta mediated by VAR2CSA phosphorylation.

These findings are interesting and relevant for the malaria community. The experiments performed and reported were thorough and convincing. In most cases, data and methods were clearly explained. The data provide the evidence needed for the authors to make their conclusions. I only have some minor points or questions that should be addressed.

Reviewer #2: The manuscript by Dorin-Semblat et al provides in vitro evidence to delineate the function of FIKK1 in phosphorylating VAR2CSA which impacts cytoadhesion property to CSA. The work described here follows a similar line of work recently published by the authors on VAR2CSA phosphorylation by PfCK2. This is a well-planned study that supports the conclusions drawn. The authors are aware of the limitations of the conclusions drawn from in vitro studies and refrained from over-interpreting their results. I recommend publication of this work with some modifications.

Specific comments:

Conclusions of this study are derived from orthogonal experiments that include, co-localization, co-IP, in vitro binding experiments, and kinase assays. Importantly, the authors demonstrate that the knockout of FIKK1 causes reduction in CSA adhesion while VAR2CSA expression remains unchanged. Authors recognize the possibility of another protein kinase (PfCK2?) co-immunoprecipitating that may contribute to VAR2CSA phosphorylation. Mass spectrometric analysis of the IP could have ruled out that possibility. Additionally, phosphoproteomic analysis of VAR2CSA in wild type vs FIKK1 cKO would have strengthened the conclusion.

It is not clear if there are any phenotypic change during the induction by rapamycin.

In Fig 6B, there are residual phosphorylation in DBL1-6 mutants, suggesting additional phosphosites may be involved.

There is a possibility that FIKK1 can mediate changes in adhesion through its functions at the erythrocyte membranes rather than exclusively through VAR2CSA phoshphorylation. Although the data presented in the manuscript supports the conclusions drawn, a robust discussion on other possibilities, such as changes erythrocyte cytoskeleton causing remodeling RBC membrane architecture, would improve the manuscript.

Reviewer #3: The current manuscript by Dorin-Semblat et al shows that the P. falciparum kinase phosphorylates the parasite ligand var2csa increasing the parasite binding affinity to csa. The study is elegantly done using a combination of molecular tools, including knockout parasites, pull down and phosphorylation assays on parasite lysates and recombinant proteins, proteomics approaches and parasite binding studies. The authors show that the phosphorylation residues on the N-terminal multidomain region of the var2csa, and that preventing phosphorylation significantly reduces the parasite binding to csa. The study is sound, logical and experiments were well controlled. Although I consider that no additional experiments are needed for publication, there were some points that would strengthen the manuscript. Although they could be considered minor, these suggestions would significantly improve the clarity of the manuscript.

**********

**Part II – Major Issues: Key Experiments Required for Acceptance**

Please use this section to detail the key new experiments or modifications of existing experiments that should be absolutely required to validate study conclusions.

Generally, there should be no more than 3 such required experiments or major modifications for a "Major Revision" recommendation. If more than 3 experiments are necessary to validate the study conclusions, then you are encouraged to recommend "Reject".

Reviewer #1: NA

Reviewer #2: None in particular.

Reviewer #3: (No Response)

**********

**Part III – Minor Issues: Editorial and Data Presentation Modifications**

Please use this section for editorial suggestions as well as relatively minor modifications of existing data that would enhance clarity.

Reviewer #1: My main comment is that the authors identified several phosphosites (such as S429, S433) as phosphorylated by FIKK1, however they were also shown to be phosphorylated by PfCK2 in their previous paper (Dorin Semblat, Plos pathogens 2025). How do the authors explain these results? Do they think that these residues are phosphorylated by both kinases? They should discuss this point.

The selection for a clone positive for VAR2CSA expression at the IE surface is not clearly explained. It would help the reader to better explain their protocol.

Interestingly, Figure 5B suggests that FIKK1 phosphorylates the endogenous VAR2CSA (band indicated with a *). However, it would be more convincing to load immuno-conjugated material (DMSO and RAPA-treated) incubated with ATP32 alone to confirm this observation.

There is a typo line 242

Reviewer #2: It is suggested to include alternative possibilities of FIKK1-mediated changes in adhesion.

Reviewer #3: - Quantification of immunofluorescences: I would suggest for good scientific practice and for better representation of experimental variability that immunofluorescences in the manuscript are quantified. This is especially important for the images shown in Figure 1C in which co-localization between var2csa and SBP1 seem to be higher than with FIKK1HA. How many iRBC were quantified in this experiment? Similarly, quantification of Figure 3A is missing, and in Figure 3E I suggest to move the FACS quantification to the main figure, as this is the most representative experiment of the phenotype. Likewise, I would clearly show that Figure 4A and B correspond to the same experiment.

- I would suggest to indicate the wall shear stress instead of flow rate, as the later is dependent on the dimensions of the channel/flow chamber. If the authors use an IBIDI set up, a conversion should be available in the vendors website.

- Clarity of figures: The figures in the manuscript are not self-explanatory and this impacts the reading of the manuscript. Simple experimental schematics or labeling the protein gels and western blot lines, instead of just numbering them will be helpful for the reader.

- PfEMP1 terminology. The var2csa structure is not shown, which might make difficult the understanding certain sections of the manuscript for non var/PfEMP1 aficionados. This is especially important for Figures 5 and 6. Also, the authors use interchangeably the term extracellular domain of VAR2CSA and rDBL1.6, this could be confusing when different terms are used in the result section and in the figure.

- Implications for future development of drugs. The unphosphorylated form of var2csa still presents cytoadhesion. Although the authors do not claim that a pan-FIKK inhibitor would prevent cytoadhesion, the reference in the text (line 407-409) appears a bit out of the blue. It could be good to add a couple of sentences to contextualize it with the results of the manuscript.

**********

PLOS authors have the option to publish the peer review history of their article (what does this mean?). If published, this will include your full peer review and any attached files.

If you choose “no”, your identity will remain anonymous but your review may still be made public.

**Do you want your identity to be public for this peer review?**  For information about this choice, including consent withdrawal, please see our Privacy Policy.

Reviewer #1: No

Reviewer #2: No

Reviewer #3: **Yes:** Maria Bernabeu

[NOTE: If reviewer comments were submitted as an attachment file, they will be attached to this email and accessible via the submission site. Please log into your account, locate the manuscript record, and check for the action link "View Attachments". If this link does not appear, there are no attachment files.]

**Figure resubmission:**

-->While revising your submission, we strongly recommend that you use PLOS’s NAAS tool (https://ngplosjournals.pagemajik.ai/artanalysis) to test your figure files. NAAS can convert your figure files to the TIFF file type and meet basic requirements (such as print size, resolution), or provide you with a report on issues that do not meet our requirements and that NAAS cannot fix.

After uploading your figures to PLOS’s NAAS tool - https://ngplosjournals.pagemajik.ai/artanalysis, NAAS will process the files provided and display the results in the "Uploaded Files" section of the page as the processing is complete. If the uploaded figures meet our requirements (or NAAS is able to fix the files to meet our requirements), the figure will be marked as "fixed" above. If NAAS is unable to fix the files, a red "failed" label will appear above. When NAAS has confirmed that the figure files meet our requirements, please download the file via the download option, and include these NAAS processed figure files when submitting your revised manuscript.

**Reproducibility:**

To enhance the reproducibility of your results, we recommend that authors of applicable studies deposit laboratory protocols in protocols.io, where a protocol can be assigned its own identifier (DOI) such that it can be cited independently in the future. Additionally, PLOS ONE offers an option to publish peer-reviewed clinical study protocols. Read more information on sharing protocols at https://plos.org/protocols?utm_medium=editorial-email&utm_source=authorletters&utm_campaign=protocols

# Author comment: Author response to Decision Letter 1

**DOI:** 10.1371/journal.ppat.1014108.r002

8 Jul 2026

Attachment Submitted filename: Answers to reviewers.docx

# Editor report: Decision Letter 1

**DOI:** 10.1371/journal.ppat.1014108.r003

**Authors:** Tracey Lamb, Leann Tilley

12 Jul 2026

Dear Dr. Gamain,

We are pleased to inform you that your manuscript 'FIKK1, a member of the FIKK kinase family, phosphorylates VAR2CSA and regulates adhesion of Plasmodium falciparum-infected erythrocytes to the placental receptor CSA' has been provisionally accepted for publication in PLOS Pathogens.

Before your manuscript can be formally accepted you will need to complete some formatting changes, which you will receive in a follow up email. A member of our team will be in touch with a set of requests.

Please note that your manuscript will not be scheduled for publication until you have made the required changes, so a swift response is appreciated.

IMPORTANT: The editorial review process is now complete. PLOS will only permit corrections to spelling, formatting or significant scientific errors from this point onwards. Requests for major changes, or any which affect the scientific understanding of your work, will cause delays to the publication date of your manuscript.

Should you, your institution's press office or the journal office choose to press release your paper, you will automatically be opted out of early publication. We ask that you notify us now if you or your institution is planning to press release the article. All press must be co-ordinated with PLOS.

Thank you again for supporting Open Access publishing; we are looking forward to publishing your work in PLOS Pathogens.

Best regards,

Leann Tilley

Academic Editor

PLOS Pathogens

Tracey Lamb

Section Editor

PLOS Pathogens

Sumita Bhaduri-McIntosh

Editor-in-Chief

PLOS Pathogens

orcid.org/0000-0003-2946-9497

Michael Malim

Editor-in-Chief

PLOS Pathogens

orcid.org/0000-0002-7699-2064

***********************************************************

Reviewer Comments (if any, and for reference):

# Editor report: Acceptance letter

**DOI:** 10.1371/journal.ppat.1014108.r004

**Authors:** Tracey Lamb, Leann Tilley

Dear Dr Gamain,

We are delighted to inform you that your manuscript, "FIKK1, a member of the FIKK kinase family, phosphorylates VAR2CSA and regulates adhesion of Plasmodium falciparum-infected erythrocytes to the placental receptor CSA," has been formally accepted for publication in PLOS Pathogens.

We have now passed your article onto the PLOS Production Department who will complete the rest of the pre-publication process. All authors will receive a confirmation email upon publication.

The corresponding author will soon be receiving a typeset proof for review, to ensure errors have not been introduced during production. Please review the PDF proof of your manuscript carefully, as this is the last chance to correct any scientific or type-setting errors. Please note that major changes, or those which affect the scientific understanding of the work, will likely cause delays to the publication date of your manuscript. Note: Proofs for Front Matter articles (Pearls, Reviews, Opinions, etc...) are generated on a different schedule and may not be made available as quickly.

Soon after your final files are uploaded, the early version of your manuscript, if you opted to have an early version of your article, will be published online. The date of the early version will be your article's publication date. The final article will be published to the same URL, and all versions of the paper will be accessible to readers.

For Research Articles, you will receive an invoice from PLOS for your publication fee after your manuscript has reached the completed accept phase. If you receive an email requesting payment before acceptance or for any other service, this may be a phishing scheme. Learn how to identify phishing emails and protect your accounts at https://explore.plos.org/phishing.

Thank you again for supporting open-access publishing; we are looking forward to publishing your work in PLOS Pathogens.

Best regards,

Sumita Bhaduri-McIntosh

Editor-in-Chief

PLOS Pathogens

orcid.org/0000-0003-2946-9497

Michael Malim

Editor-in-Chief

PLOS Pathogens

orcid.org/0000-0002-7699-2064
